# Evaluating a Psychoeducation Program to Foster Chinese Primary School Students’ Covitality

**DOI:** 10.3390/ijerph18168703

**Published:** 2021-08-18

**Authors:** Min Fang, Li Zhang, Dachen Pan, Jiashu Xie

**Affiliations:** 1 Cognition & Human Behavior Key Laboratory of Hunan Province, Department of Psychology, Hunan Normal University, Changsha 410081, China; faumin@foxmail.com (M.F.); 202070090786@hunnu.edu.cn (L.Z.); panDAchen9301@163.com (D.P.); 2 Public Course Teaching and Research Office, Hunan Nursing School, Changsha 410600, China

**Keywords:** covitality, school belonging, bullying victimization, life satisfaction, growth psychoeducation program

## Abstract

This investigation evaluated the Growth Psychoeducation Intervention (GPI) designed to increase primary school students’ covitality, a construct describing the beneficial combinatorial effects of positive psychological skills and mindsets. Students with higher covitality levels have stronger relationships with their teachers and classmates, and behave in more positive ways. This GPI intervention study employed a pretest-posttest-follow quasi-experimental design to evaluate a culturally adapted group counseling intervention designed to foster Chinese senior primary school students’ (*n* = 189, ages 9–12 years) covitality levels. The hypothesis was that covitality increases would positively correlate with school belonging and life satisfaction and less frequent bullying victimization. The Social Emotional Health Survey-Primary (SEHS-P) assessed the effectiveness of the GPI eight-week program to promote mental health and decrease bullying. GPI demonstrated effectiveness by improving students’ covitality and school belonging and reducing bullying victimization.

## 1. Introduction

Chinese senior primary school students are in the crucial childhood-to-adolescence developmental stage as they matriculate into junior middle school. During this stage, adolescent psychological and mental health levels are closely related to their current learning and life quality, and crucial to their subsequent adaptation to adolescence, a significant transition period of children’s psychological development [1]. Contemporary China is experiencing social change and rapid economic expansion. Accompanying these social forces, children experience increasingly fierce competition during the primary school to junior high transition [2]. As a result, Chinese senior primary school students are increasingly expected to engage in academic tasks independently. Managing these developmental tasks can shape children’s well-being by increasing anxiety and depression, and even extend into adulthood with impaired social, familial, and academic functioning [3]. In addition to academic competition, school bullying has become a prominent factor affecting the mental health of senior pupils. In one survey [4], a considerable proportion (2.6–37.6%) of Chinese children and teenagers have committed or suffered bullying behavior. Bullying endangers students’ physical and mental health [5], especially the victims of bullying tend to have depression, anxiety, lower self-esteem, loneliness, and even suicidal ideation, and continue to affect individual physical and mental health in later life. Unfortunately, whether on-campus or off-campus, in China psychological services are minimal [6].

When available Chinese interventions employ treatment models designed to prevent the emergence of psychological disorders. These interventions are often untested and created by teachers, counselors, or psychologists. These programs aim to improve mental health generally but are not grounded in a theoretical model. Most Chinese intervention studies only focus on a specific positive trait. A few Chinese psychoeducation studies report (a) significant improvements in personality dimensions, coping styles, and self-esteem when the intervention focused on positive psychological skills and traits [7,8,9]; and (b) significant reduction in depression levels [10]. Acknowledging a gap between Chinese senior primary students’ psychological development challenges and the available professional services, the development and validation of culturally relevant psychoeducational intervention models are essential.

### 1.1. Positive Psychoeducation Approaches

Gratitude [11], optimism [12], and hope-benefit finding [13] are among various psychological traits positively correlated with adolescents’ academic performance and life satisfaction. Moreover, child resilience research shows that the effects of cumulative risk and cumulative assets are powerful predictors of mental health and development, more so than any single risk or positive trait. In the past 15 years, positive psychology research has used cumulative factor methods to detect the impact of multiple and co-occurring character strengths or strengths on young people’s subjective well-being, life satisfaction, and other outcome variables [14]. School psychologists have long recognized the potential benefits of fostering students’ well-being, eschewing a traditional deficit emphasizing mental health model [15]. Instead, they are committed to applying prevention and intervention services informed by balanced complete mental health models [16] within a comprehensive, multitier school psychological services framework [17]. Within this positivistic school psychology lens, Furlong et al. proposed covitality as a construct that refers to positive mental health resulting from the synergy of multiple positive psychological skills and mindsets [18]. Covitality has similarities with the Chinese culturally relevant integrative psychological Suzhi construct. Psychological Suzhi is a widely applied Chinese construct describing a comprehensive and integrated set of psychological qualities of Chinese children and adolescents, psychological suzhi also emphasizes the combined effect of individual components [19].

### 1.2. Covitality Model

Covitality has been used as a comprehensive indicator to evaluate students’ social and emotional health [18,20,21,22]. The covitality construct for primary school students comprises four core positive psychological traits—gratitude, zest, optimism, and persistence. Traditionally, gratitude is the sense of appreciation experienced by someone who has received a material gift or benefits from another person. Because gratitude is closely related to establishing, maintaining, and strengthening teacher–student relationships, Furlong et al. [18] suggested that gratitude is as a keystone positive-psychological trait for schools to cultivate in youth. Optimism is one of the critical traits of positive psychology; some have defined optimism as an explanatory style for conceptualizing experiences of defeat or failure [23]. Furlong et al. [18] define optimism as a general attitude toward the future, characterized by a consistent expectation of good or positive outcomes [24]. Zest is approaching and experiencing life with excitement and energy. It is a positive psychological characteristic of young people who are motivated and promising [25,26]. Finally, persistence encompasses persistence and passion directed to accomplishing one’s goals [27,28]. Because the present study’s participants were in primary school, persistence does not involve long-term effort. Hence, persistence refers to students’ ability to perform tasks that require some endurance or continuous performance for some time. In the covitality model, gratitude, zest, and optimism consider past, present, and future time perspectives, respectively. These three positive psychological orientations predict well-being [29]. Persistence is a primary factor predicting academic achievement [30]. The theoretical conception of covitality has been supported in empirical studies in the United States, Japan, South Korea, Australia, and other countries [31], as well as in China [32].

### 1.3. Effectiveness of Covitality Intervention Programs

A review of the previous literature found that the results provided by previous interventions were not particularly optimistic. Although not statistically significant, the covitality score increased over time, suggesting that they have practical significance in promoting effective social interventions and assessments in the real world [33,34,35]. In addition, the main problems encountered in the effectiveness of these programs have been related to their implementation. Specifically, students have doubts about the credibility of a program when those who present it are external to the context of the situation. Furthermore, the SEHS-P (Furlong et al., 2013) has not yet been validated as a progress monitoring tool that is sensitive to change over time; and therefore, may not be conducive to producing the effect sizes that are necessary to show statistical significance [18].

### 1.4. Culturally Relevant Covitality Fostering Psychoeducation Program

The GPI was guided by the transtheoretical model and stages of change (TTM) [36], a psychological perspective widely used in health behavior interventions. The Transtheoretical Model and Stages of Behavioral Transitions Change (TTM) was proposed by Prochaska in the early 1980s and has become a widely used behavioral change theoretical model [36]. TTM was initially used as an intervention study for smoking cessation and later developed into various areas, including alcohol and substance abuse, dietary behavior, sedentary lifestyle, HIV prevention, compliance behavior, unplanned pregnancy intervention, and other behavioral problems [37]. Its advantage is that it divides a simple behavior into five specific stages to explain how the motivation and needs of different people’s behaviors play a vital role in the prediction, prevention, and intervention of strategies.

The TTM divides human behavior change into five stages: precontemplation stage, contemplation stage, preparation stage, action stage, and maintenance stage [37].

‘Precontemplation’ is the stage in which people do not intend to take action in the near term, usually measured as the next six months. The outcome interval may vary, depending on the behavior. People may be in this stage because they are uninformed or under-informed about the consequences of their behavior. Or they may have tried to change a number of times and become demoralized about their abilities to change.

In ‘contemplation’, people intend to change their behaviors in the next six months. They are more aware than precontemplators of the pros of changing but are also acutely aware of the cons. This balance between the costs and benefits of changing can produce profound ambivalence and keeps people stuck in contemplation for long periods of time. This phenomenon is often characterized as chronic contemplation or behavioral procrastination. These folks also are not ready for traditional action-oriented programs that expect participants to take action immediately.

In ‘preparation’, people intend to take action soon, usually measured as the next month. Typically, they already have taken some significant step toward the behavior in the past year. They have a plan of action, such as joining a health education class, consulting a counselor, talking to their physician, buying a self-help book, or relying on a self-change approach. These are the people who should be recruited for action-oriented programs, such as traditional smoking-cessation or weight-loss clinics.

People in the ‘action’ stage have made specific, overt modifications in their lifestyle within the past six months. Because action is observable, behavior change often has been equated with action. Typically, not all modifications of behavior count as action in this model. In most applications, people have to attain a criterion that scientists and professionals agree is sufficient to reduce risks for disease.

‘Maintenance’ is the stage in which people have made specific, overt modifications in their lifestyles and are working to prevent relapse, but they do not apply change processes as frequently as people in action. They are less tempted to relapse and are increasingly more confident that they can continue their changes.

The change of health behavior needs to step by step from the last stage to the next, and finally reach the goal of behavior change. The TTM contains 10 steps.

1. Consciousness raising: finding and learning new facts, ideas, and tips that support the healthy behavior change.
2. Dramatic relief: experiencing the negative emotions (fear, anxiety, worry) that go along with unhealthy behavioral risks.
3. Self-reevaluation: realizing that the behavior change is an important part of one’s identity as a person.
4. Environmental reevaluation: realizing the negative impact of the unhealthy behavior or the positive impact of the healthy behavior on one’s proximal social and/or physical environment.
5. Self-liberation: making a firm commitment to change.
6. Helping relationships: seeking and using social support for the healthy behavior change.
7. Counterconditioning: substitution of healthier alternative behaviors and cognitions for the unhealthy behavior.
8. Contingency management: increasing the rewards for the positive behavior change and decreasing the rewards of the unhealthy behavior.
9. Stimulus control: removing reminders or cues to engage in the unhealthy behavior and adding cues or reminders to engage in the healthy behavior.
10. Social liberation: realizing that the social norms are changing in the direction of supporting the healthy behavior change.

## 2. Materials and Methods

### 2.1. Study Purpose

Given the shortcomings of mental health preventive and early intervention in Chinese schools, there is a pressing need to develop and evaluate culturally relevant psychoeducation interventions. Based on the unified development theory [38] and the ecosystem theory [39], this study designed a targeted intervention program to improve the covitality of senior students in primary school, which directly targets their social and emotional health. The primary aim was to evaluate the effectiveness of the Chinese culturally relevant Growth Psychoeducation Intervention (GPI) to reduce bullying victimization and enhance covitality.

### 2.2. Ethical Concerns

This study was approved by the Research Project Ethical Review Board at Hunan Normal University. The study was presented to the local education authority and obtained approval from the authority’s administrators. The authority obtained informed consent from the parents. At the end of the study, the results were presented to the authority’s administrators and to the teachers, children, and parents.

### 2.3. GPI Rationale and Development

The GPI group psychological counseling process aims to (a) improve covitality by integrating individuals into the group counseling process and (b) provoke students learning of specific healthy behaviors and beliefs. TTM incorporates into group psychological counseling, making coaching goals clear. The change of healthy behavior in the TTM is essentially a change in health beliefs [40]. Previous studies have found that all dimensions of primary school students’ covitality (gratitude, zest, optimism, and persistence) are directly related to healthy behaviors [28,41,42,43], so the theory of healthy behavior change also guides the intervention research of primary school students’ covitality.

**Figure 1:**
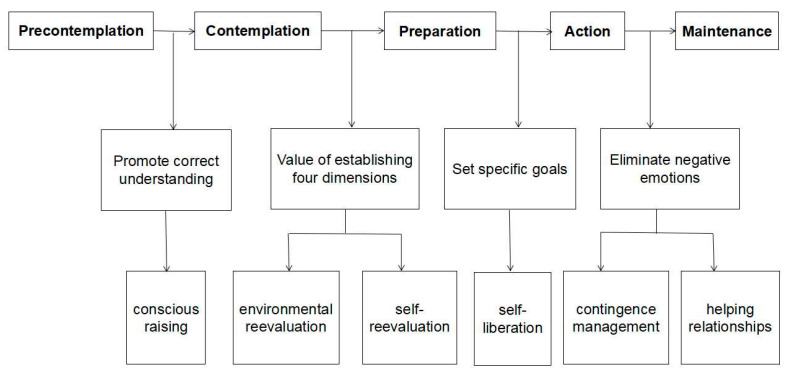
Flowchart of design of group counseling intervention program.

For example, considering gratitude intervention research on primary school students the intervention, guided by the TTM, takes the perception of the consequences of gratitude behavior, emotional experience, and environmental reevaluation as the first step to transition the intervention object from the pre-contemplation to the contemplation. This is accomplished by exploring personal goals and self-reevaluation. In this process, the student transitions from contemplation to preparation, and finally, through social relationship support and contingence management to help the individual transition from preparation to action. According to the 10 steps and 5 stages of the TTM, the study suggests that the intervention of the positive attributes of the social and emotional health of primary school students can be structured to improve the awareness of positive attributes of social and emotional health. The research suggests that the intervention of the positive attributes of elementary school students’ social and emotional health can be divided into the following stages: (a) enhancing the awareness of positive attributes of social and emotional health; (b) establishing the value of positive attributes; (c) establishing specific action plans and plans targeting positive attributes; (d) eliminating negative emotion; and (e) establishing an excellent collective atmosphere. The specific intervention design process is shown in Figure 1. The GPI psychoeducation training approach and lesson content are shown in Table 1.

### 2.4. Participants

The present study employed a quasi-experimental design with repeated measures (pretest, posttest, and follow-up) and a control group. The sample came from one elementary school in Changsha City, Hunan Province, China. Before implementing the psychoeducation intervention, all students in Grades 3 to 6 completed a pretest survey to assess their social and emotional health. Student responses to the pretest survey identified entire class groups with relatively low social and emotional health indicators and entire classes with no significant differences. Since the current investigation evaluated a psychoeducation program designed for use with intact class groups, and was conducted in an applied school context, random assignment to treatment and control groups was not feasible. Consequently, consultation with the school’s psychology teachers, teaching directors, and headteachers identified an intact class group with a balanced gender and heterogeneous pretest scores to receive the group counseling intervention. The control group classes had no significant differences on the pretest measures compared with the GPI intervention classes. The psychoeducation intervention was implemented in general public schools: one third-grade class (*n* = 42) and one fifth-grade class (*n* = 46) with one third-grade (*n* = 51) and one fifth-grade (*n* = 50) control group. Students ranged between 9–12 years, 54% identifying as male.

### 2.5. Measures

#### Quantitative Measures

Social and Emotional Health Scale-Primary (SEHS-P)—The SEHS-P (adapted for Chinese students) [32] assesses elementary school children’s mindsets associated with positive development. The measure used in the present study has 21 items assessing four covitality components (gratitude zest, optimism, and persistence). It uses a six-point point response scale: 1 = never, 2 = almost never, 3 = sometimes, 4 = often, 5 = very often, and 6 = always like this. A sample optimism item is: “I usually expect to have a good day” (see www.covialityucsb.info, accessed on 16 March 2017). Although originally validated with samples of California school children, the SEHS-P has been adapted and validated in other international educational contexts [29,44,45]. For the current investigation, the SEHS-P alpha reliabilities were as follows: gratitude (0.85), zest (0.85), optimism (0.80), and covitality composite (0.94). Students’ covitality is obtained by adding up the scores of gratitude, zest, optimism, and persistence in the four subscales. Higher scores reflected higher levels of covitality.

Delaware Bullying Victimization Scale (DBVS)—The DBVS (adapted for Chinese students) [46,47] is a 13-item measure frequency of student bullying victimization. It employs a six-point scale (1 = never, 2 = occasionally, 3 = once or twice a month, 4 = once a week, 5 = many times a week, 6 = every day). Sample items are: I was teased by someone saying hurtful things to me, and I was pushed or shoved on purpose. A higher score indicates a student’s frequent, serious bullying victimization. For the current investigation, the DBVS alpha reliability was 0.93.

Psychological Sense of School Membership Scale (PSSM)—The PSSM (adapted for Chinese students) [48] has 18 items divided with two subscales: Belonging (13 items) and Rejection (5 items). The items are rated using a six-point Likert-type scale (1 = totally disagree, 2 = disagree, 3 = basically disagree, 4 = basically agree, 5 = agree, 6 = totally agree). Sample items are: I feel like a part of my school, and I can really be myself at my school. Higher scores indicate a better school membership. For the current investigation, the DBVS alpha reliability for both subscales was (0.82).

### 2.6. Procedures

For the pretest, posttest, and follow-up assessments, students were informed that their responses would be confidential and used for research purposes only. The parents of third- and fifth-grade children were informed of the study, and positive consent was requested for their child’s participation. All third- and fifth-grade students who were present the day of data collection completed the study measures. Students responded using a paper-and-pencil format. During the investigation, whole-class response patterns identified intervention and control groups classes.

One week after the pretest (April 2017), the intervention program was implemented in treatment groups during the school day and within the school schedule (May–September 2017). The posttest was collected one week after the past GPI lesson, and the follow-up was administered in February 2018. One experimenter presented the intervention with two assistants from Hunan Normal University with a practical educational experience and an understanding of the theoretical basis for the group psychological counseling approach. The eight psychoeducation units were presented in 90-min session.

### 2.7. Statistical Analysis

First, we conducted an independent sample *t*-test for the Grade 3 and Grade 5 treatment groups and their respective control group to evaluate overall pretest differences. Second, an independent sample *t*-test evaluated the pretest-posttest change for GPI treatment and control groups, with a paired sample *t*-test comparing each grade’s treatment-control scores. Third, to evaluate the stability of the group counseling effects, an independent sample *t*-test examined follow-up survey scores for each grade’s experimental and control groups, with a paired sample *t*-test providing a comparative analysis of the follow-up test and the posttest for each grade’s treatment class. Lastly, a repeated measure ANOVA and simple main-effect analysis compared bullying victimization and student belonging measures for the treatment and the control groups. It was carried out using the IBM SPSS statistical software 22.0 (IBM, Armonk, NY, USA) was used to analyze the data.

## 3. Results

### 3.1. Pretest Differences

The independent sample *t*-test of the pretest responses found no statistically significant differences between experimental and control groups in any of the variables studied.

### 3.2. Covitality Comparisons

A paired-sample *t*-test comparing GPI treatment and control pretest and posttest scores (Table 2) found several significant changes for the covitality total score and its four components. For the third-grade GPI treatment class, all SEHS-P posttest scores were significantly higher than the pretest scores, with large effect size differences (d > 0.80). For the fifth-grade GPI treatment group, there is a significant difference between the posttest covitality, gratitude, zest, and optimism scores with moderate or high effects sizes (d > 0.50). The difference in the persistence dimension scores was marginally significant. The control group classes had no significant pretest-posttest differences on any SEHS-P scores.

The students’ scores from the five-month follow-up are shown in Table 3. The difference in either the covitality total score and its components were not significant, suggested the intervention effect had positive and persistent effects. In addition, the GPI experimental class covitality levels remained relatively high, indicated that the intervention effects were sustained.

### 3.3. Bullying Victimization Comparisons

**Figure 2:**
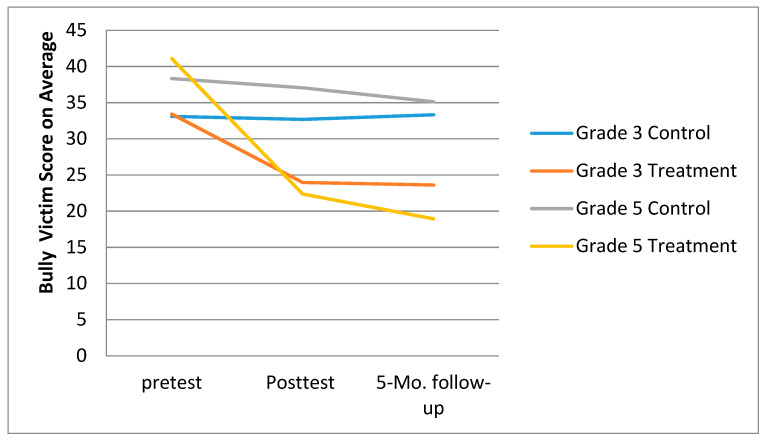
Comparison of bullying victimization scores at three time points for third- and fifth-grade students.

The Grade 3 treatment and the control class bullying victimization scores are shown in Figure 2. A repeated measures ANOVA found significance for (a) the Time effect, *F* = 7.72, *p* < 0.01, η^2^_p_ = 0.078; (b) Group effect, *F* = 8.99, *p* > 0.01, η^2^_p_ = 0.09; and (c) the interaction between Time and Group, *F* = 7.00, *p* > 0.01, η^2^_p_ = 0.071. The simple effect analysis showed that for the control class (a) there was no statistically significant difference in the bullying victim scores at three time points, *F* = 0.035, *p* > 0.05, η^2^_p_ = 0.001; (b) the difference in the amount of delay time at the three time points in the experimental class was statistically significant, *F* = 7.97, *p* < 0.01, η^2^_p_ = 0.268. The pretest bullying victim score (33.42 ± 11.26) was higher than the posttest (23.95 ± 10.15, *p* < 0.01) and follow-up (23.61 ± 11.42, *p* < 0.01) scores.

The Grade 5 treatment and the control class bullying victimization scores are shown in Figure 2. The analysis found significance for the (a) Time effect, *F* = 66.63, *p* < 0.01, η^2^_p_ = 0.414; (b) Group effect, *F* = 82.38, *p* < 0.01, η^2^_p_ = 0.47; and (c) the interaction between Time and Group, *F* = 41.22, *p* < 0.01, η^2^_p_ = 0.31. The simple effect analysis showed that (a) there was no statistically significant difference in the bullying victim scores of the control class at three time points, *F* = 1.932, *p* > 0.05, η^2^_p_ = 0.04; and (b) the GPI treatment class had statistically significant lower bullying victim scores at three time points, *F* = 82.14, *p* < 0.01, η^2^_p_ = 0.64. The bullying victimization pretest score (41.08 ± 6.85) was higher than the posttest (22.36 ± 7.61, *p* < 0.01) and follow-up (28.93 ± 8.82, *p* < 0.01) scores.

### 3.4. School Belonging Comparisons

**Figure 3:**
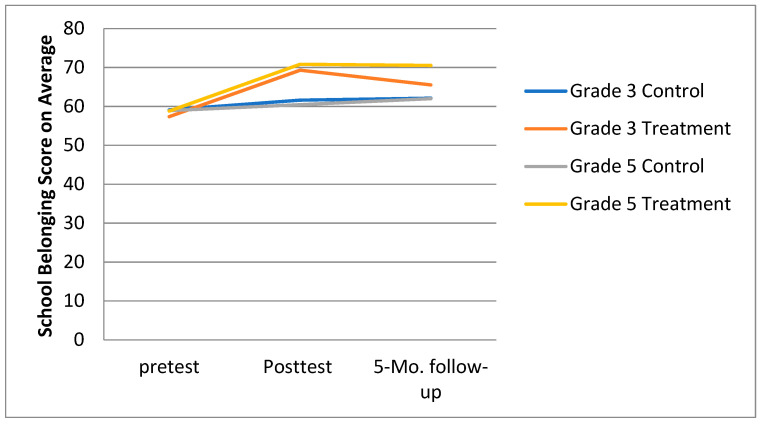
Comparison of sense of school belonging scores at three time points for third- and fifth grade students.

Figure 3 shows the Grade 3 school belonging scores at pretest, posttest, and follow-up assessments. A repeated measures analysis of variance found (a) a significant time effect, *F* = 17.87, *p* < 0.01, η^2^_p_ = 0.16; (b) the group effect was not significant, *F* = 1.98, *p* > 0.05, η^2^_p_ = 0.02; however; (c) the interaction between Time and Group was significant, *F* = 6.88, *p* < 0.01, η^2^_p_ = 0.07. The simple effect analysis showed that (a) there was no statistically significant difference in control class’ school belonging scores across the three time points, *F* = 1.82, *p* > 0.05, η^2^_p_ = 0.39; and (b) the GPI treatment class had statistically significant different student belonging scores across the three time points, *F* = 26.63, *p* < 0.01, η^2^_p_ = 0.37. The pretest school belonging score (57.35 ± 13.65) was lower than the posttest (69.28 ± 10.83, *p* < 0.01) and follow-up (65.52 ± 11.05, *p* < 0.01) scores.

Figure 3 shows the Grade 5 school belonging scores at pretest, posttest, and follow-up assessments. A repeated measures analysis of variance results found significance for the (a) Time effect, *F* = 26.08, *p* < 0.01, η^2^_p_ = 0.29; (b) Group effect, *F* = 13.83, *p* < 0.01, η^2^_p_ = 0.13; and the interaction between Time and Group, *F* = 12.13, *p* < 0.01, η^2^_p_ = 0.12. A simple effect analysis showed that there was no statistically significant difference in the sense of school belonging scores for the control class at three time points, *F* = 1.70, *p* > 0.05, η^2^_p_ = 0.04. The GPI treatment class’ student belonging scores were statistical different at the three time points, *F* = 30.04, *p* < 0.01, η^2^_p_ = 0.40). The pretest school belonging score (58.78 ± 5.94) was lower than the posttest (70.80 ± 10.35, *p* < 0.01) and follow-up (70.52 ± 10.89, *p* < 0.01) scores.

## 4. Discussion

The present study showed statistically significant differences between children who received the GPI intervention and those who did not participate in the program. The GPI group-based psychoeducation program produced improvements in the treatment group participants documented by significant (a) increases in covitality, (b) increases in student belonging, and (c) reductions in bullying victimization. Analyses of these data from the pilot study provide promising, preliminary support about durable increases in positive affect and covitality, with moderate to large effect sizes (Cohen’s d). This is different from previous studies [33,35]. Ana Martínez-Martínez’s study showed that after the implementation of the intervention, the reduction effect of bullying behavior was decreased, while the social and emotional ability did not change significantly [35]. The possible reason is that the GPI in this study is based on the TTM combined with the ecosystem theory. Using the GPI to innovate and develop research. The activities in each unit are designed according to the principles and methods of the TTM, which makes the purpose of intervention more clear and the effect more obvious. After the group counseling intervention, children experienced more frequent positive emotions and greater contentment with themselves, friends, and their living environment. Five months after the intervention concluded, the improvements in covitality were maintained. Treatment group students and a significant increase in student belonging and reducing bullying victimization also became apparent. A possible explanation for these results may be that group counseling interventions based on the TTM increased students awareness of the importance of positive traits and willingness to make changes [40].

The current study results are consistent with other studies showing the efficacy of covitality to improve school performance with other Asian culture children and adolescents [49]. The possible reasons are as follows. First, the GPI group counseling intervention improved the treatment class students’ covitality and sense of school belonging contributing to a more positive classroom climate and an associated reduction of bullying behavior. Previous reports also found that covitality can effectively predict Asian culture students’ campus adaptation [50]. Second, the GPI based on the TTM increased students’ awareness of the importance of positive traits and their willingness to make changes. Studies have shown that the gratitude dimension of covitality has a significant positive correlation with the sense of belonging to school. Teenagers with high gratitude level also have a higher sense of belonging to school. Meanwhile, people with high gratitude level pay more attention to the establishment and maintenance of their interpersonal relationship. With high levels of gratitude teenagers are more sensitive to feel the classmates and teachers’ help and support, at the same time, it shows more grateful cognitive, emotional, and behavioral support and promote the students and teachers [51]; as a result, the GPI effectively changed the students from the cognitive understanding of individual positive qualities, will further improve the assessment of students on campus of belonging. There is a significant negative correlation between bullying victimization and school belonging, and a positive school belonging can build a stronger prosocial peer relationship, thus reducing the participation rate of school bullying [52,53]. Furthermore, the insignificant difference in the scores of the persistence dimension of the fifth-grade GPI class may be due to the low difficulty of a persistence dimension unit intervention activity—Difficult Event Table. Feedback from students after the activity shows that the game is not difficult at all, there is no challenge, and it does not meet the requirements of the event design; hence, it might not have provoked persistence awareness as intended.

The results show that the GPI experimental class is significantly higher than the control class except persistence, the total score of covitality and other dimensions. The reason why the persistence dimension is not significant may be that some games set in group counseling program are too simple, which does not conform to the age characteristics of the fifth grade students.

As to why to have the “standing Junzi” activity for the group counseling program: in China, soldiers are a group of people with noble beliefs, strong will, and dedication spirit. They are not afraid of hardship or tiredness when serving the people, which is a good example for us to learn from. According to “National Defense Education Law of the People’s Republic of China” and “Military Training Teaching Outline of Senior Middle Schools”, China’s senior high schools have formulated military training work for students before enrollment to promote the improvement of students’ comprehensive quality [54,55]. Considering that the group’s supplementary target is primary school students, we only let students watch the military documentary “Daily Sniper” and experience the standing Junzi in military training. The film shows the firm will and hard work of Chinese soldiers. Through watching this film, students can feel the steadfast quality of soldiers improve their self-awareness and understand that if you persist in doing things, you can win. Standing Junzi is a kind of training to exercise people’s willpower and cultivate persistence quality. It requires people to maintain a standard action for a long time, even if tired, but also to adhere to the end of the instruction. Through the theme activity of “Standing Junzi”, we will learn to persist emotionally and improve our self-efficacy, so as to improve our determination to face difficulties in life and study.

## 5. Practical Implications

Social and emotional health is a growing perspective proposed by western school psychology to study individual positive mental health. Primary school students are in a critical stage of rapid physical and psychological development and changes. Research on the development of social and emotional health of students at this stage has important practical significance for Chinese student support services. The findings of this GPI effectiveness study complement Chinese education’s principles and practices. A high-quality Chinese education seeks to cultivate emotional attitudes and values, emphasizing students’ comprehensive quality and improvement [56]. Challenging training is a crucial point of quality education [57]. The emphasis on challenging is particularly evident in physical education classes that purposefully present strenuous exercises to improve students’ persistence. The current study developed and evaluated the effectiveness of the standardized GPI psychoeducation for Chinese primary school students. This is a new resource for front-line teachers and counselors to increase understanding and fostering of students’ social and emotional health.

## 6. Limitations

The results of this study should take into account the following limitations. First, the sample size was small, mainly composed of primary school students from one school in Changsha City, limiting the generalizability of the findings. Future randomized control trials are needed to evaluate GPI’s effectiveness before broad dissemination into Chinese schools. Second, the intervention did not consider how varying school contexts and climates might influence pupils’ activities. Research shows that there are differences between the mental health of primary school students in public and private schools [58], and school climate explains approximately 30–50% of the between school variance in children’s mental health outcomes [59]. In our study, the intervention did not consider how varying school contexts and climates might influence pupils’ mental health. Third, although the GPIO treatment group and the control group were from the same school, there was strict control to exclude cross-group contamination. Finally, although we arranged the activities of other groups in the control group, we did not set a blank group, so the GPI did not completely exclude the placebo effect, that is, the study was insufficient.

## 7. Conclusions

Senior students in primary school are in a critical developmental period, and nurturing their positive mental health affects their immediate academic progress and influences future psychological development. We plan to expand the research scope in future research and include more implementations and different life quality outcome indicators. In addition, considering that both school and family have a substantial impact on pupils, future research can consider intervening pupils’ covitality from the perspective of the family environment. It could also consider training teachers to implement the scheme and assessing whether they can deliver equally promising results, providing a more sophisticated approach to mental health education in schools.

## Figures and Tables

**Table 1:** Growth psychoeducation intervention (GPI) session topics and content overview.

Sessions Topic	Session Content and Title
Getting reacquainted, building mutual trust and security	Strong Winds Blow The Rules and Principles
2. Improve gratitude consciousness	Love at the Fingertips ^1^ Gratitude Heart
3. Learning to appreciate and praise	You are Good at Playing Basketball! Guess Who He or She Is Reveal Your True Feelings I Admire You
4. Learning to be optimistic	Thousand-hand Kwan Yin Be an Optimistic Person Make Every Day Full of Joy
5. Emotion management	I See Create an Emotional Calendar ^2^
6. Learning to help others	Giving is the Best Return Through the Virgin Forest Our Road to Friendship It’s Good to Have You Around
7. Improving persistence	Forrest Gump Difficult Event Table
8. Finding self-worth	Thousand Knots Daily Sniper ^3^ My Junzi is the Most Standard ^4^

Examples of GPI Lessons: ^1^ Love at the Fingertips: one’s four fingers represent a certain action. Sticking out a finger is putting your face to one side, indicating that you don’t want to know each other yet. Extending two fingers is to shake each other’s right hand, on behalf of willingness to get to know each other. Extending three fingers is a warm handshake that shows you like the other person and is happy to meet him or her. Holding out four fingers is a warm hug that shows a willingness not only to get to know each other but also to be good friends. ^2^ Create an Emotional Calendar: Record your mood for a month. Write the date in your book and use different colors to represent how you are feeling today. Green means happy, black means sad, and red means angry. ^3^ Daily Sniper: A documentary film that conveys to students the strong character of soldiers. The documentary shows the soldiers’ uncompromising spirit to complete tasks in tough conditions, such as Standing Junzi for three hours, running 10 km with 10 kg load and wearing a cotton-padded jacket in the sun for an afternoon in a weather of 40 degrees, which often requires great persistence. ^4^ Standing Junzi: Standard posture requirements are as follows: (1). the heels together, the foretoe is separated about 60 degrees, the legs are tight and straight, but also need to lift the hip, stomach, chest, head up; (2) The arms are naturally drooping. The thumbs of both hands are placed at the second joint of the index finger; (3) the jaw slightly closed, eyes look forward; (4) Lean forward slightly with your weight on the palm of your front foot. This training aims to strengthen patriotism education and enhance national defense awareness. Exercise seek to develop tenacious willpower and physical fitness, collective consciousness and cultivate the spirit of individuality, and cultivate the spirit of hardship and develop a good work and rest. A main aim is to cultivate students’ persistence.

**Table 2:** Pretest and posttest measures for experimental and control groups.

		Pretest		Posttest				
		M	SD	M	SD	*t*	*p*	d
Gratitude	Gr 3 control	22.02	5.59	22.22	5.16	0.19	0.849	0.04
	Gr 3 experimental	21.89	5.26	25.39	4.11	3.07	0.004	**0.67**
	Gr 5 control	22.29	3.95	21.74	4.49	0.74	0.465	0.23
	Gr 5 experimental	22.02	4.40	25.80	4.17	2.69	0.010	**0.63**
Optimism	Gr 3 control	21.00	5.26	20.20	4.86	−0.73	0.471	0.15
	Gr 3 experimental	19.03	5.37	24.51	4.21	4.79	0.000	**1.02**
	Gr 5 control	22.63	4.47	21.79	5.20	0.99	0.327	0.16
	Gr 5 experimental	21.95	4.73	24.90	4.27	3.47	0.001	**0.62**
Zest	Gr 3 control	23.62	6.79	23.38	6.86	−0.17	0.867	0.04
	Gr 3 exp	23.31	5.87	28.23	5.28	3.54	0.001	**0.84**
	Gr 5 control	22.94	5.71	22.85	6.25	0.07	0.942	0.01
	Gr 5 experimental	24.65	5.81	27.62	6.54	2.07	0.045	0.51
Persistence	Gr 3 control	24.16	5.38	23.40	5.60	−0.68	0.498	0.14
	Gr 3 experimental	22.97	4.74	27.57	3.24	4.92	0.001	**0.97**
	Gr 5 control	25.18	4.37	25.10	4.61	0.24	0.811	0.02
	Gr 5 experimental	24.50	4.58	26.42	4.82	1.82	0.076	0.42
Covitality	Gr 3 control	90.80	19.42	89.20	19.65	−0.39	0.699	0.08
	Gr 3 experimental	87.08	14.62	105.50	13.26	5.47	0.001	**1.26**
	Gr 5 control	92.56	14.19	91.25	16.07	0.57	0.575	0.08
	Gr 5 experimental	93.77	15.95	104.92	17.30	2.95	0.005	**0.70**

Note: Cohen’s d values in bold represent moderate and large effects size differences.

**Table 3:** Posttest and 5-Mo. Follow up test measures for experimental and control groups.

		Posttest		5-Mo. Follow Up Test				
Variable	Experimental Group	M	SD	M	SD	*t*	*p*	d
Gratitude	Grade 3	25.41	4.06	24.40	4.32	−0.84	0.405	0.23
	Grade 5	25.97	4.08	24.46	6.20	−1.71	0.096	0.37
Optimism	Grade 3	24.51	4.21	23.57	3.95	−1.31	0.198	0.22
	Grade 5	24.77	4.25	23.66	5.51	−1.24	0.222	0.26
Zest	Grade 3	28.23	5.28	27.05	5.36	−1.63	0.111	0.22
	Grade 5	27.71	6.60	26.15	7.14	−1.23	0.230	0.24
Persistence	Grade 3	27.49	3.30	26.24	3.66	−1.91	0.064	0.34
	Grade 5	26.50	4.80	25.60	5.65	−1.04	0.305	0.19
Covitality	Grade 3	105.64	12.81	101.26	13.43	−1.87	0.069	0.33
	Grade 5	105.44	17.26	100.75	22.73	−1.07	0.294	0.27

Note: All posttest and 5-Mo. follow up test comparisons represent small effect size difference, except for Grade 5 Persistence.

## Data Availability

Data available on request due to restrictions e.g., privacy or ethical. The data presented in this study are available on request from the corresponding author. The data are not publicly available due to the privacy and specificity of the people involved in the study.
